# MicroRNA-205 promotes cell invasion by repressing TCF21 in human ovarian cancer

**DOI:** 10.1186/s13048-017-0328-1

**Published:** 2017-05-05

**Authors:** Jun Wei, Lahong Zhang, Jennifer Li, Shuguang Zhu, Minghui Tai, Clifford W. Mason, Julia A. Chapman, Evelyn A. Reynolds, Carl P Weiner, Helen H Zhou

**Affiliations:** 1 Department of Obstetrics and Gynecology, University of Kansas Medical Center, Kansas City, Kansas 66160 USA; 2 Department of Clinical Laboratory, The Affiliated Hospital of Hangzhou Normal University, Hangzhou Zhejiang, 310036 People’s Republic of China

**Keywords:** microRNA(miR)-205, TCF21, Ovarian Cancer

## Abstract

**Background:**

Ovarian cancer is the leading lethal, gynecological malignancy in the United States. No doubt, the continued morbidity and mortality of ovarian cancer reflects a poor understanding of invasive mechanisms. Recent studies reveal that ovarian cancers express aberrant microRNAs (miRNAs or miRs), some of which have oncogenic or tumor suppressor properties. Several studies suggested that miR-205 is involved in tumorigenesis. Presently, we investigate the molecular mechanisms and target of miR-205 in ovarian cancer.

**Methods:**

Quantitative real-time polymerase chain reaction and western blot were performed to assess miR-205 and transcription factor 21 (TCF21) expression in ovarian cancer and normal ovary samples. The effect of miR-205 on TCF21 was determined by luciferase reporter assay and western blot. The effect of miR-205 and TCF21 on cell invasion was quantitated using transwell invasion assay.

**Result:**

miR-205 expression was increased in ovarian cancer and it promoted the invasive behavior of ovarian cancer cell lines (OVCAR-5, OVCAR-8 and SKOV-3). miR-205 directly targeted TCF21, which was significantly decreased in ovarian cancer tissue. miR-205 inhibited TCF21 expression and as a consequence blunted the inhibitory effect of TCF21 on cell invasion. Matrix Metalloproteinases (MMPs) play an important role in cancer invasion and metastasis. TCF21 inhibited MMP-2 and MMP-10 and decreased ovarian cancer cell invasion. Co-transfection of TCF21 expression plasmid with miR-205 mimic diminished the inhibitory effect of TCF21 on MMP-2 and MMP-10 in ovarian cancer cells.

**Conclusion:**

miR-205 appears to have an important role in the spread of ovarian cancer by targeting TCF21. These findings offer a new mechanism of ovarian cancer tumorigenesis, which could be useful for the development of new therapeutic approaches to ovarian cancer treatment.

## Background

While ovarian cancer is the fifth leading cause of death from cancer in women, it is the number one lethal, gynecological malignancy in the United States [1]. Despite extensive clinical and basic research, only 27% of patients with advanced-stage ovarian cancer survive 5 years after their initial diagnosis. The high mortality of this disease reflects the relative absence of early symptoms and the high percentage (>60%) of patients diagnosed at an advanced stage [1]. There is an urgent need to understand the mechanisms of ovarian cancer invasion and metastasis to foster the development of targeted therapeutic strategies.

MicroRNAs (miRNAs or miRs) are small non-coding RNA molecules of 20-25 nucleotides that regulate gene expression by targeting one or more messenger RNAs (mRNAs) for translational repression or cleavage. Instead of being translated into protein, mature single-stranded miRNA binds to mRNAs to interfere with the translational process. Nearly one-third of the encoded genes in mammalian cells are regulated by miRNA [2, 3]. miRNAs have important roles in cellular processes such as development, differentiation, cell cycle, apoptosis, metabolism, and proliferation [4, 5]. Abnormal miRNA expression has been found in numerous human malignancies using oligonucleotide miRNA microarray and RT-PCR technologies [6]. About half of the human miRNAs are located in cancer-associated genomic regions that are frequently amplified, deleted, or rearranged in cancer. This suggests miRNAs have the potential to function as either tumor suppressors or oncogenes, and play a major role in cancer tumorigenesis and metastasis [6].

Several microRNA profiling studies demonstrate aberrant expression of microRNAs in ovarian cancer [7, 8]. Most of these microRNAs, including miR-199a and miR-125b, were down-regulated in ovarian cancer tissues compared to non-cancer tissues. Other microRNAs such as miR-200a, miR-200c and miR-141 were upregulated [7, 8]. Human miR-205 is located on chromosome 1 and the effect of cancer on its expression is tissue and type specific. Multiple investigators have reported that miR-205 is up-regulated in lung, kidney and bladder cancers [9, 10], but down regulated in prostate, esophageal, melanoma, and breast cancers [11–14]. In prostate cancer, miR-205 re-expression induces the tumor suppressor genes IL24 and IL32 by targeting specific sites in their promoter regions [11]. We found that miR-205 expression was significantly increased and TCF21 (a tumor suppressor gene) significantly decreased in epithelial ovarian carcinomas compared with normal ovary. TCF21 has been predicted using computational methods to be a potential target gene of miR-205. In this investigation, we test the hypothesis that miR-205 regulates TCF21 mediated tumor suppression in ovarian cancer leading to tumor progression.

## Methods

### Clinical specimens

Thirty (30) ovarian cancer samples and 12 normal ovary controls were collected from women undergoing surgery at University of Kansas Medical Center. All samples were from epithelial ovarian carcinomas, including 21 serous ovarian carcinomas and 9 mucinous ovarian carcinomas (8 stage I, 7 stage II, 7 stage III, 8 stage IV). The average age of the cancer patients was 57.9 years, while the average age of women providing normal ovaries was 58.5 years. The tissues were collected intraoperatively and snap-frozen in liquid nitrogen immediately after collection, then stored at -80 °C for future analysis. Documented, written consent was obtained from patients as approved by the human subjects committee at the time. This study was approved by Institutional Review Board (Human Subjects Committee) of University of Kansas Medical Center. The IRB ID is CR00000581 for Study 12,995.

### Quantitative reverse-transcription PCR (qRT-PCR)

Total RNA was extracted using Trizol™ (Life Technologies) according to the manufacturer’s instructions. RNA purity and RNA concentrations were confirmed using NanoDrop™ (NanoDrop Technologies). cDNA was generated from 1 μg of total RNA using Omniscript™ RT kit (Qiagen) or NCode™ miRNA First-Strand cDNA Synthesis Kit (Life Technologies) according to standard procedures. Real-time qPCR to assess mRNA and miRNA expression was performed using SYBR green on the IQ5 Real-Time PCR thermocycler (Bio-Rad). Threshold cycle (Ct) values were calculated according to the iQ5 real-time detection software. Gene-specific primers are listed in Table 1.

**Table 1:** Primers used in Real-time qRT-PCR

Primer Name	Sequence (5′ to 3′)	Accession Number
TCF21 Forward	CAGATCCTGGCTAACGACAAA	NM_198392
TCF21 Reverse	CCACTTCTTTCAGGTCACTCTC	
MMP2 Forward	AAGTGGTCCGTGTGAAGTATG	NM_ 004530
MMP2 Reverse	GGTATCAGTGCAGCTGTTGTA	
MMP7 Forward	GAGTGAGCTACAGTGGGAACA	NM_ 002423
MMP7 Reverse	CTATGACGCGGGAGTTTAACAT	
MMP9 Forward	TGTACCGCTATGGTTACACTCG	NM_ 004994
MMP9 Reverse	GGCAGGGACAGTTGCTTCT	
MMP10 Forward	TTGGTCACTTCAGCTCCTTTC	NM_ 002425
MMP10 Reverse	CAACAGCATCTCTTGGCAAATC	
miR-205 Forward	TCCTTCATTCCACCGGAGTCTG	MIMAT0000266
miR-205 Reverse	Universal primer from Life Technologies	

Values were normalized to 18 s rRNA or U6 small nuclear (sn) RNA. The relative amount of mRNA or miRNA in each sample was calculated using the 2^-ΔΔCT^ method [15] The results were expressed as fold change in the cancer group compared to control group.

### Western blot analysis

Protein was extracted from tissue or cells with ice-cold lysis buffer (150 mM NaCl, 1% Igepal Ca- 630, 0.5% sodium deoxycholate, 0.1% SDS, 1 mM EDTA, 50 mM Tris, pH 7.8) containing protease and phosphatase inhibitor cocktails (Sigma). Protein concentration was quantified using the Bradford™ reagent (Bio-Rad). Cleared lysates containing equivalent amounts of total protein (40 μg) were loaded onto a 15% acrylamide gel and electrophoresed in Tris/Glycine/SDS running buffer (Bio- Rad). Proteins were then transferred to polyvinylidene difluoride membranes with Tris/Glycine transfer buffer (Bio- Rad). Membranes were blocked in 2% non-fat milk and incubated overnight at 4 °C with the anti-TCF21 (1:500, Thermo Scientific-Pierce), anti- β-actin (1:500, Abcam). Primary antibodies were visualized using horseradish peroxidase-labeled species-appropriate IgG (1:5000 or 1:10,000; GE Healthcare Life Sciences) and enhanced chemiluminescence (GE Healthcare Life Sciences). Western blot results were captured by ChemiDOC Imaging System™ (Bio-Rad) and quantified by Image Lab Software (Bio-Rad).

### TCF21 expression plasmid construction

The TCF 21 gene (NM_198392; 3249 bp; CDS 278-817) was amplified by PCR using specific forward: CCACGACTCTGGGAGTG (18-35), and specific reverse: GGAATCCTGCGTGCCAT (3122-3139) primers. The purified PCR product (3121 bp) was inserted into pcDNA™3.1D/V5-His-TOPO vector according to the manufacturer’s protocol (Life Technologies) and transformed into competent cells. The presence of the insert was confirmed by Hind III and XhoI double restriction enzyme digestion. The fidelity of cloned sequence was confirmed by DNA sequencing.

### Cell culture and transfection

The OVCAR-5 and OVCAR-8 cell lines were kindly donated by Dr. Qi Chen [16]. SKOV-3 was obtained from the American Type Culture Collection. Cells were cultured in DMEM (OVCAR-5) or RPMI medium1640 (OVCAR-8 and SKOV-3) with 10% fetal bovine serum and 1% penicillin/streptomycin (10,000 U) under standard cell culture conditions (37 °C, 5% CO_2_). For transfection, cells were seeded at 0.5 × 10^6^ cells per well in 6 well plates and cultured for 24 h. The synthetic miR-205 mimic (Qiagen, final concentration 50 nM), miR-205 inhibitor (Qiagen, final concentration 100 nM) and TCF21 plasmid (2.5 μg per well) were co-transfected into OVCAR-5, OVCAR-8 and SKOV-3 cells using Lipofectamine 2000™ (Life Technologies) 10 μl/well. The cells were harvested for RNA and protein isolation after 24 h incubation.

### Construction of reporter gene and luciferase reporter assay

The TCF21 segment containing target site (2593-2600) of miR-205 or mutant TCF21 segment were PCR amplified using Hotstar HiFidelity™ Polymerase kit (Qiagen). Purified PCR products were digested with NheI and XbaI and directionally inserted downstream of the firefly luciferase reporter gene in the pmirGLO Dual-Luciferase™ miRNA target expression vector (Promega). The construct insert was sequenced and verified. Ovarian cancer cells were seeded at 0.5 × 10^5^ cells per well in 24 well plates. miRNA-205 mimic (50 nM) (Qiagen), TCF21 reporter vector or mutant vector (0.25 μg), and the internal control pRL reporter vector (0.002 μg) were transfected into ovarian cancer cell lines using Lipofectamine 2000™ (Life Technologies). The cells were collected after 24 h incubation. The luciferase activity was measured using Dual-Luciferase Assay (Promega) and normalized with Renilla™ luciferase.

### In vitro invasion assay

OVCAR-5, OVCAR-8 and SKOV-3 cells were seeded in 6 well plates at 0.5 × 10^6^ cells per well with DMEM or RPMI medium1640 and 10% FBS for 24 h. Synthetic miR-205 mimic, miR-205 inhibitor or TCF21 expression plasmid were transfected into the cells with Lipofectamine 2000™ (Life Technologies). After 24 h, cells were harvested by trypsinization and counted. The invasive potential of the OVCAR-5, OVCAR-8 and SKOV-3 cells was evaluated using matrigel invasion chambers from BD Biosciences. The synthetic miR-205 mimic, miR-205 inhibitor, or TCF21 expression plasmid transfected cells in 500 μl of serum-free culture medium were placed on the matrigel-coated upper cell insert chambers (24-well inserts; pore size, 8 mm). Next, 750 μl medium containing 10% fetal bovine serum was placed in the lower chambers to serve as a chemoattractant. After 72 h of incubation at 37 °C and 5% CO_2_, cells that migrated through the matrigel onto the lower surface of the insert chambers were fixed and stained with 0.5% crystal violet (Sigma). The lower surface of the insert chambers was photographed with the ×10 objective using an inverted microscope (NIKON 80i). The numbers of cells were counted using image software.

### Statistical analysis

Statistical comparisons were performed using Student’s t test. *P* values below 0.05 were considered statistically significant. Data are presented as mean ± S.E.M.

## Results

### miR-205 expression increased in ovarian cancer tissue and ovarian cancer cell lines

**Fig. 1:**
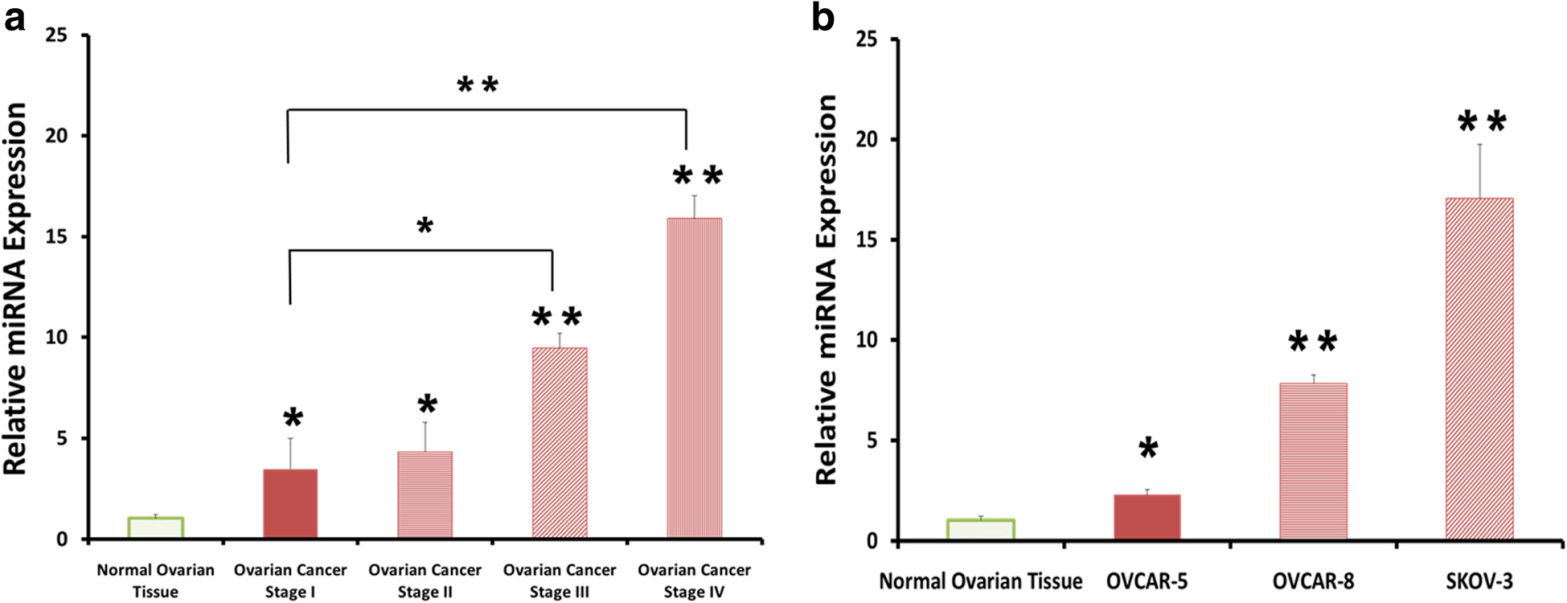
miR-205 expression was significantly up-regulated in ovarian cancer tissue and cell lines. **a** Quantitative Reverse-Transcription PCR analysis of miR-205 expression levels in normal ovarian tissue and ovarian cancer tissue stage I-IV (Stage I *n* = 8; StageII *n* = 7; Stage III *n* = 7; Stage IV *n* = 8). **b** The relative expression levels of miR-205 in three ovarian cancer cell lines compared with normal ovarian tissue. Data presented as mean ± S.E.M, * *P* < 0.05 and ** *P* < 0.01

We investigated the expression level of miR-205 in ovarian cancer and normal ovarian tissues using SYBR-Green qRT-PCR analysis. Our results showed that miR-205 expression was significantly up-regulated in stage I to stage IV ovarian cancer tissue: 3.5-fold higher in stage I; 4.3-fold higher in stage II; 9.5 -fold higher in stage III; and 15.9 -fold higher in stage IV. There was no significant difference of miR-205 expression between stage I and stage II ovarian cancer, but miR-205 expression significantly increased in stage III and stage IV compared with stage I ovarian cancer (Fig. 1a). We further confirmed the miR-205 expression in three ovarian cancer cell lines: OVCAR-5, OVCAR-8 and SKOV-3. The expression of miR-205 significantly increased in three ovarian cancer cell lines compared with normal ovarian tissue: 2.5-fold higher in OVCAR-5; 7-fold higher in OVCAR-8; and 17-fold higher in SKOV-3 (Fig. 1b). The results suggest that upregulated miR-205 in ovarian cancer tissue and cell lines may correlate with ovarian cancer carcinogenesis.

### miR-205 promotes ovcar-5, ovcar-8 and skov-3 cell invasion in vitro

**Fig. 2:**
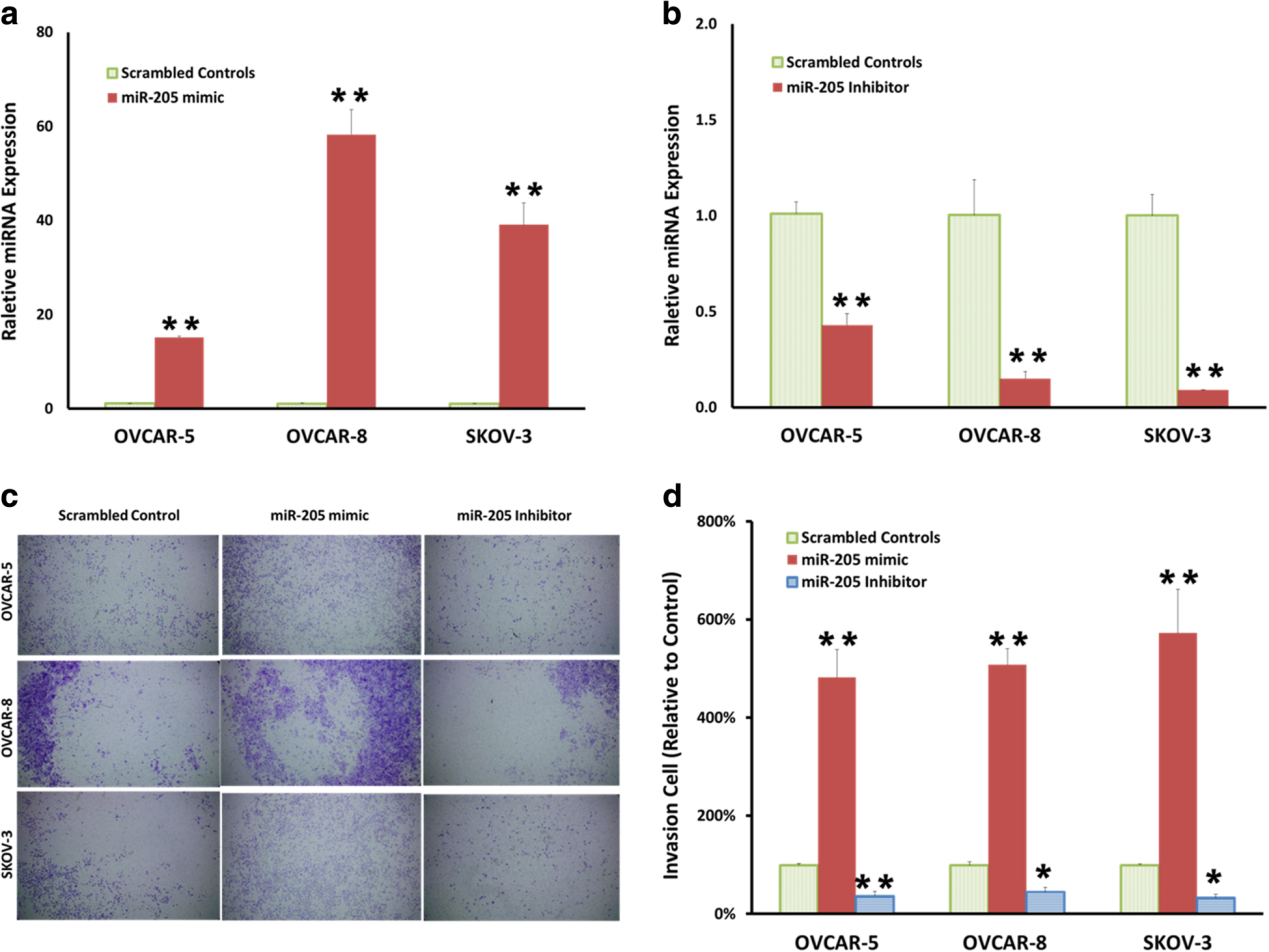
miR-205 upregulation promotes ovarian cancer cells invasion in vitro**. a** Quantitative Reverse-Transcription PCR demonstrating expression of miR-205 in OVCAR-5, OVCAR-8 and SKOV-3 cells after miR-205 mimic transfection. **b** miR-205 expression in OVCAR-5, OVCAR-8 and SKOV-3 cells after miR-205 inhibitor transfection. **c** Photograph result of invasive cells on Transwell membrane after transfection with scrambled control, miR-205 mimic or miR-205 inhibitor. **d** Quantitative analysis of OVCAR-5, OVCAR-8 and SKOV-3 invasion. Data presented as mean ± S.E.M, * *P* < 0.05 and ** *P* < 0.01

miR-205 mimic and miR-205 inhibitor were transfected into OVCAR-5, OVCAR-8 and SKOV-3 cells and subjected to Transwell invasion assays to assess whether the invasive properties were modified by transfection. miR-205 expression was significantly increased after transfection with the miR-205 mimic in three cell lines compared with scrambled controls (Fig, 2a). In contrast, it was decreased after miR-205 inhibitor transfection (Fig. 2b). The mean number of cells penetrating the Transwell membrane was significantly increased 5 to 6-fold by the miR-205 mimic compared to scrambled controls while the miR-205 inhibitor significantly decreased the number of cells (2 to 3-fold) crossing the membrane (Fig. 2c-d). These findings suggest miR-205 promotes ovarian cancer cell invasion and acts as a tumor oncogene in ovarian cancer tumorigenesis.

### miR-205 directly targets TCF21 and represses TCF21 expression in ovarian cancer cells

**Fig. 3:**
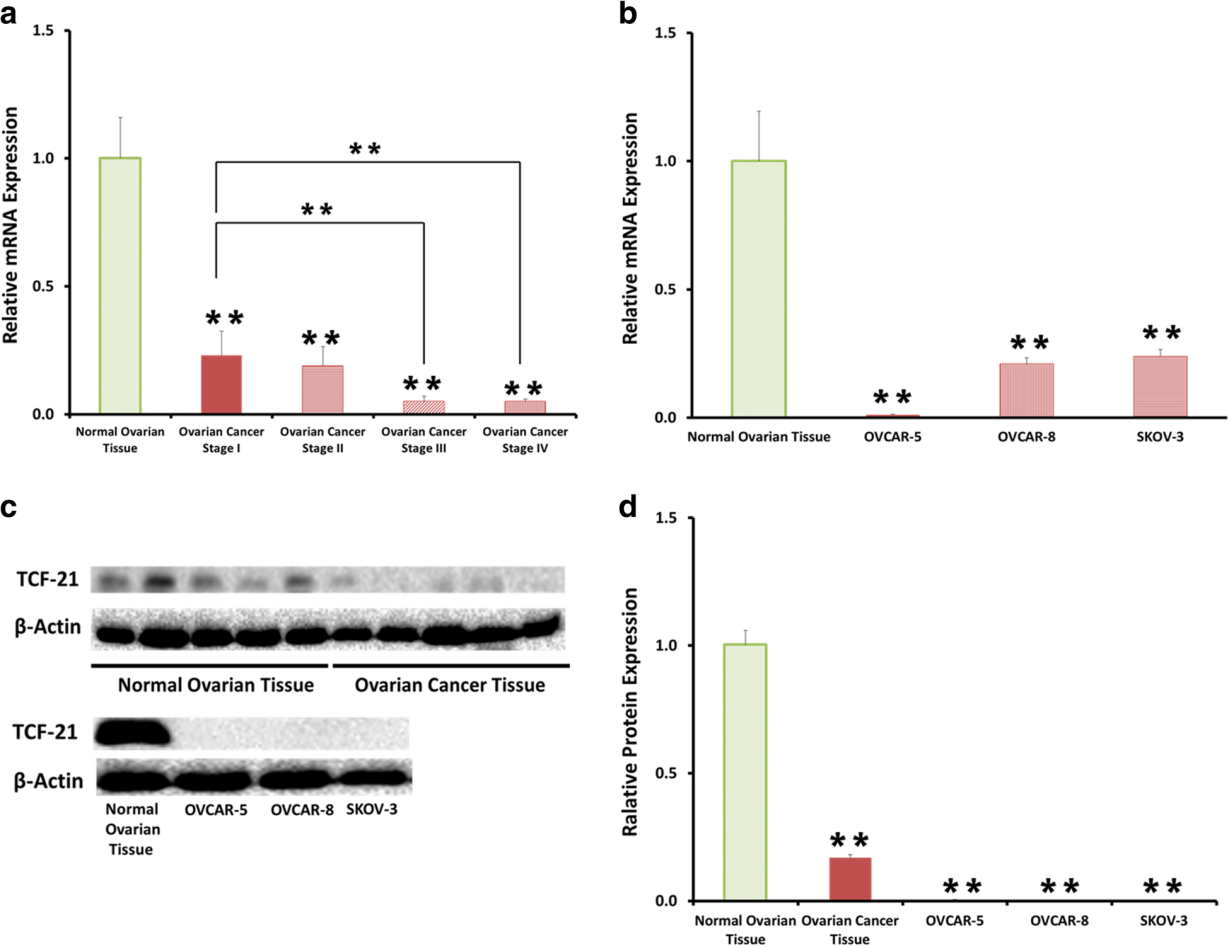
TCF21 expression is significantly downregulated in ovarian cancer tissue and cell lines. **a** Quantitative reverse-transcription PCR results showing TCF21 expression levels in stage I to stage IV ovarian cancer tissue and normal ovarian tissue. **b** Quantitative reverse-transcription PCR result showing TCF21 expression levels in ovarian cancer cell lines OVCAR-5, OVCAR-8 and SKOV-3 compared to normal ovarian tissue. **c** Western blot showing TCF21 protein expression in ovarian cancer tissue and ovarian cancer cell lines OVCAR-5, OVCAR-8 and SKOV-3 compared to normal ovarian tissue. **d** Quantitative analysis of protein levels of TCF21 normalized to β-actin. Data presented as mean ± S.E.M, *P < 0.05 and **P < 0.01

Using bioinformatics software tools, we found that miR-205 targets TCF21. We used qRT-PCR to quantitate TCF21 expression in ovarian cancer tissue and ovarian cancer cell lines. TCF21 expression was significantly down-regulated in stage I to stage IV ovarian cancer tissue compared to normal ovarian tissues. There was no significant difference of TCF21 expression between stage I and stage II ovarian cancer, but TCF21 expression significantly decreased in stage III and stage IV compared to stage I ovarian cancer (Fig. 3a). TCF21 expression was significantly decreased in three ovarian cancer cell lines OVCAR-5, OVCAR-8 and SKOV-3 compared to normal ovarian tissues (Fig. 3b). Using Western blot analysis, we found that TCF21 protein levels were significantly decreased in ovarian cancer tissue and ovarian cancer cell line OVCAR-5, OVCAR-8 and SKOV-3 compared with normal ovarian tissue (Fig. 3c-d).

**Fig. 4:**
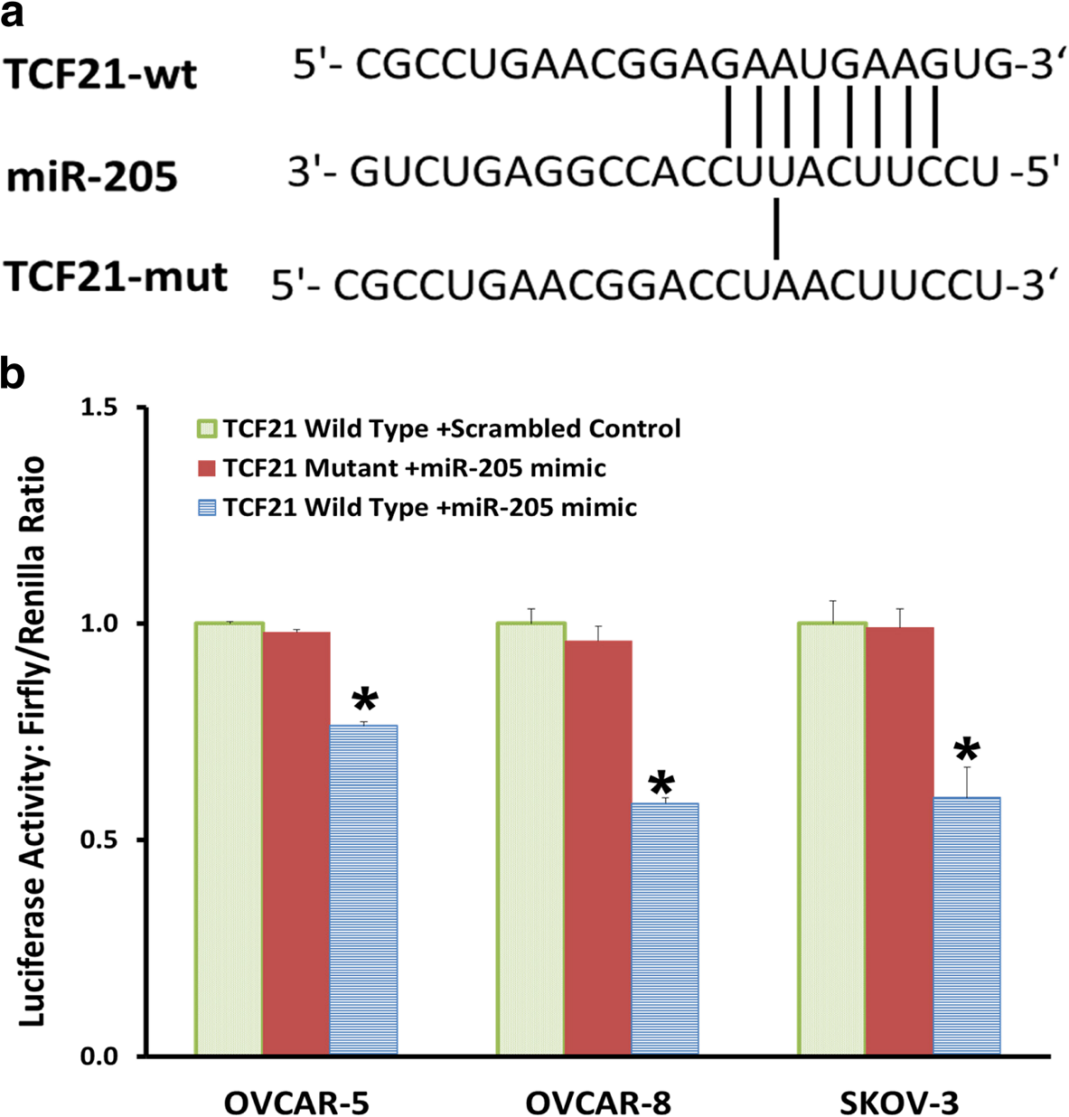
miR-205 directly targets TCF21**. a** Algorithms predicted TCF21 contains miR-205 binding site on its 3′-untranslated regions. **b** Dual-luciferase assay revealed the direct interaction between miR-205 mimic and TCF21 reporter vector in OVCAR-5, OVCAR-8 and SKOV-3 cells. Data presented as mean ± S.E.M, *P < 0.05 and **P < 0.01

To confirm the targeting of TCF21 by miR-205, we performed a luciferase reporter assay. The TCF21 segment containing the target site of miR-205 or mutant 3’UTR sequences were inserted downstream of the firefly luciferase reporter gene (Fig. 4a). miR-205 mimic was co-transfected into ovarian cancer cell lines along with the TCF21 wild-type or mutant reporter vectors and internal control pRL reporter vectors. Transfection efficiency was normalized using Renilla luciferase. The Dual-Luciferase Assay revealed that the luciferase activity of TCF21 reporter vector was suppressed by transfection of the miR-205 mimic (Fig. 4b).

**Fig. 5:**
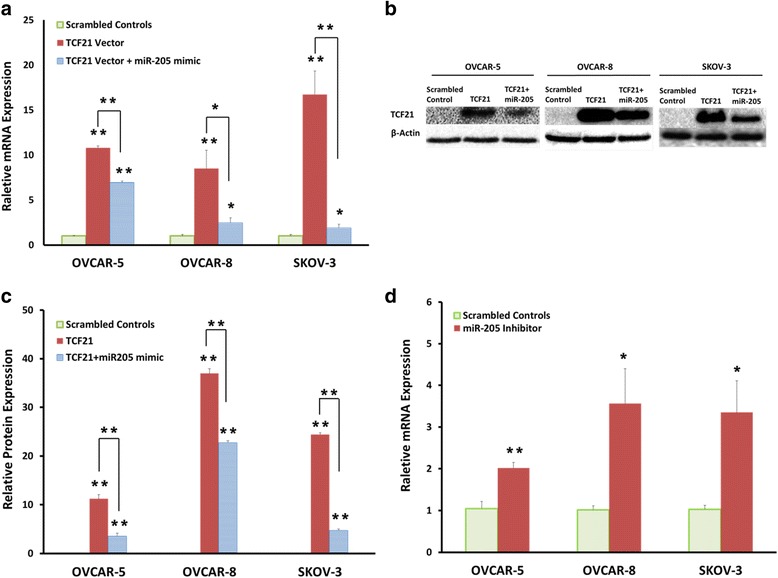
miR-205 represses TCF21 expression in ovarian cancer Cells. **a** Quantitative reverse-transcription PCR result showing TCF21 expression after TCF21 expression plasmid and miR-205 mimic co-transfection in OVCAR-5, OVCAR-8 and SKOV-3 cells. **b** The Western blot illustrating TCF21 protein levels after TCF21 expression plasmid and miR-205 mimic transfection in three ovarian cancer cell lines. **c** Quantitative analysis of relative protein levels of TCF21 normalized to β-actin. **d** Quantitative reverse-transcription PCR result showing TCF21 expression after miR-205 inhibitor transfection in OVCAR-5, OVCAR-8 and SKOV-3 cells. Data presented as mean ± S.E.M, *P < 0.05 and **P < 0.01

To further confirm miR-205 regulates TCF21 expression, we transfected TCF21 expression vectors, miR-205 mimic and miR-205 inhibitor into OVCAR-5, OVCAR-8 and SKOV-3 cells. Changes in TCF21 expression were analyzed by qRT-PCR and Western blot. As expected, transfection of TCF21 expression plasmid significantly increased both TCF21 mRNA expression (Fig. 5a) and protein levels in ovarian cancer cell lines (Fig. 5b-c). However, TCF21 expression was significantly decreased when miR-205 mimic was co-transfected with TCF21 expression plasmid in the OVCAR-5, OVCAR-8 and SKOV-3 cells (Fig. 5a-c). miR-205 inhibitor significantly increased TCF21 mRNA expression in the OVCAR-5, OVCAR-8 and SKOV-3 cells (Fig. 5d). However, TCF21 protein expression didn’t show significant difference compared with control after miR-205 inhibitor transfection (data not shown). These results indicate that miR-205 directly targets TCF21 and represses TCF21 expression in ovarian cancer.

### miR-205 attenuated the Inhibition of TCF21 mediated cell invasion in vitro

**Fig. 6:**
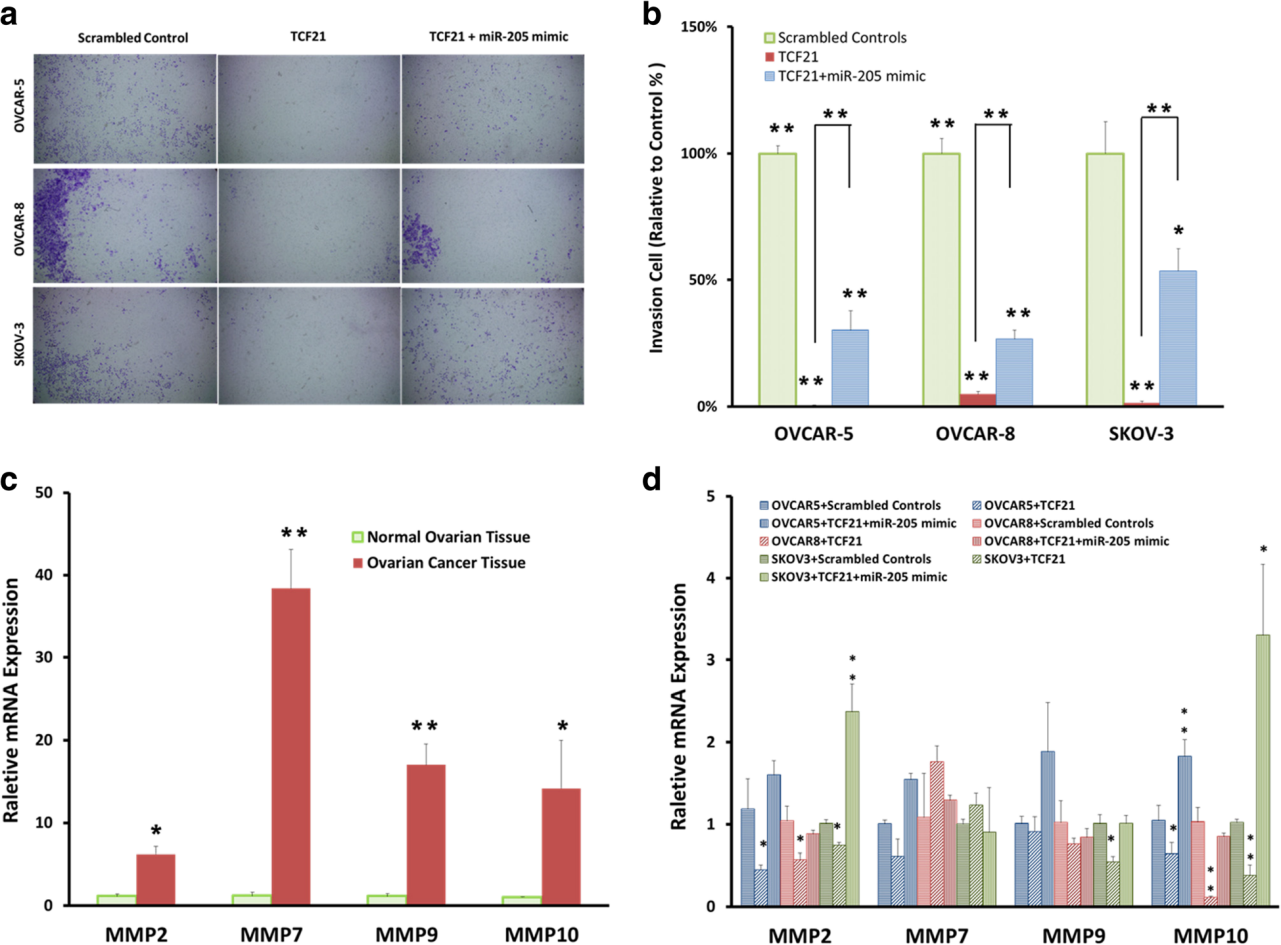
TCF21 inhibits ovarian cancer cell invasion and miR-205 attenuates its inhibition of cell invasion **(a)** Photographs of invasive cells on Transwell membrane after transfection with TCF21 expression plasmid and 205-mimic. The experiments were performed at the same time with miR-205 inhibitor (Fig. 2c) and therefore, the same scrambled controls were used in the analysis of invasion. **b** Quantitative analysis of invasion viability. **c** Quantitative reverse-transcription PCR result showing MMP-2, MMP-7, MMP-9 and MMP-10 expression levels in ovarian cancer tissue and normal ovarian tissue. **d** Quantitative reverse-transcription PCR result showing MMP-2, MMP-7, MMP-9 and MMP-10 expression levels in OVCAR-5, OVCAR-8 and SKOV-3 cells after transfection with TCF21 expression plasmid and miR-205 mimic. Data presented as mean ± S.E, *P < 0.05 and **P < 0.01

We overexpressed TCF21 in OVCAR-5, OVCAR-8 and SKOV-3 cells to determine whether or not TCF21 alters the invasion properties of ovarian cancer cells. Using Transwell invasion assay, we found that TCF21 decreased the invasion properties of OVCAR-5, OVCAR-8 and SKOV-3 cells compared to control cells (Fig. 6a-b). These findings support the conclusion that TCF21 is a tumor suppressor gene in ovarian cancer cells and restoring TCF21 in ovarian cancer cells inhibits their invasive properties.

We then co-transfected TCF21 expression plasmids and miR-205 mimic into OVCAR-5, OVCAR-8 and SKOV-3 cells, and performed invasion assays to test the direct impact of miR-205 on the inhibition of cell invasion properties mediated by TCF21. Co-transfection TCF21 expression plasmid with miR-205 mimic attenuated the TCF21-mediated inhibition of cell invasion (Fig. 6a-b). These results demonstrate that miR-205 promotes ovarian cancer cells invasion by targeting TCF21 in ovarian cancer cells.

### miR −205 regulates TCF21-mediated Inhibition of cell invasion through MMPs

Matrix metalloproteinases (MMPs) are requisites for degrading components of the extracellular matrix (ECM) in tumor cell invasion. Our results showed that MMP-2, MMP-7, MMP-9 and MMP-10 expression were significantly up-regulated in ovarian cancer tissue compared with normal ovarian tissues (Fig. 6c). To better understand the mechanism of miR-205 and TCF21 on ovarian cancer cells invasion properties, we tested the hypothesis that MMPs were important for invasive behavior. Up-regulation of TCF21 reduced MMP-2, MMP-9, and MMP-10 expression in OVCAR-5, OVCAR-8 and SKOV-3 cells (Fig. 6d). When co-transfected with miR-205 mimic and TCF21 expression plasmids, the expression of MMP-2 was unchanged in OVCAR-5 and OVCAR-8 cells, but increased in SKOV-3 cells. The expression of MMP-10 was also unchanged following co-transfection in OVCAR-8 cells, but increased in OVCAR-5 and SKOV-3 cells. The expression of MMP-9 was unchanged in SKOV-3 cells (Fig. 6d). The inhibition of MMPs may be one mechanism by which TCF21 decrease ovarian cancer cell invasion properties. This is supported by our findings that miR-205 targets TCF21 and reverses TCF21-mediated decreases in ovarian cancer cells invasion.

## Discussion

miRNAs regulate diverse molecular pathways and can function as tumor suppressors and/or oncogenes by targeting specific mRNAs. Recent evidence suggested that miRNAs expression profiles differ among cancer types such as esophageal cancer [13], lung cancer [17], breast cancer [18], prostate cancer [19], colon cancer [20], pancreatic cancer [21], and ovarian cancer [22, 23]. Other studies showed that over-expression or repression of miRNAs is tissue-specific and may possess opposing functions in different types of cancer. For example, miR-205 inhibited cancer cell proliferation and invasion in prostate, esophageal, melanoma, and breast cancer [11–14, 24–27], but promoted tumorigenesis in lung and cervical cancer [28, 29]. In the present study, we found that miR-205 expression was up-regulated in all stages (I – IV) of ovarian cancer tissue and ovarian cancer cell lines compared to normal ovary. miR-205 expression levels were associated with ovarian cancer stages, and increased significantly in stage III and stage IV ovarian cancer compared with stage I and II ovarian cancer. The increases of miR-205 in ovarian cancer cells: OVCAR-5, OVCAR-8 and SKOV-3 enhanced their invasive behavior. These findings are consistent with miR-205 being a tumor oncogene in ovarian cancer with important roles in tumor invasion and metastasis.

The TCF21 gene is located on chromosome 6q23 and encodes a basic, helix–loop–helix transcription factor that is essential for epithelial cell differentiation [30, 31]. TCF21 is expressed in numerous tissues including lung, gut, gonad, urinary tract, spleen and kidney [30]. Epigenetic-induced loss of TCF21 expression is a common event in human malignancies [32–34]. TCF21 is silenced in squamous cell carcinomas, non-small-cell lung cancer cells, and clear cell renal cell carcinoma [31, 35]. TCF21 acts as a tumor suppressor gene and a decrease in TCF21 resulted in increased undifferentiated mesenchymal cells, which in turn increased cancer cell migration [36]. Tumors lacking TCF21 expression were two to three times larger and more vascular than TCF21*-*positive tumors [31]. One explanation for this behavior is that the loss of TCF21 decreases the activation of the known metastatic suppressor, KISS1, causing an increase in the metastatic potential of the tumor [21, 33, 37]. TCF21 expression is downregulated in metastatic melanoma by hypermethylation of a promoter, and overexpression of TCF21 inhibits the motility of melanoma cells via KISS1 [2, 33]. The present findings strongly support a role for TCF21 in cell invasion properties of ovarian epithelial cancers. We demonstrated that TCF21 expression was downregulated in stage I to stage IV ovarian cancer tissue and ovarian cancer cell lines. Decreased TCF21 expression levels were related to ovarian cancer stages. There were significantly decreased TCF21 expression in stage III and stage IV ovarian cancer compared with stage I and II ovarian cancer. Forced expression of TCF21 in OVCAR-5, OVCAR-8 and SKOV-3 cells decreased their invasive properties. These results are consistent with the function of TCF21 as a tumor suppressor by inhibiting invasion in ovarian cancers.

Two prior studies concluded that TCF21 is targeted by miR-21 and miR-224 [38, 39]. In this study, we provide evidence that TCF21 is also modulated by miR-205. We identified an inverse relationship of increased miR-205 expression and decreased TCF21 expression in ovarian cancer tissue which was more prominent in the advanced stages of the disease. We further confirmed our hypothesis in three ovarian cancer cell lines: OVCAR-5, OVCAR-8 and SKOV-3. Increasing miR-205 in ovarian cancer cells caused a significant reduction in TCF21 activity as measured using a luciferase reporter construct containing the miR-205 target site. Real time qRT-PCR and Western blot analysis confirmed that miR-205 in ovarian cancer cells down-regulated TCF21 expression. This was further supported by the findings that decreased miR-205 expression with miR-205 inhibitor up-regulated TCF21 mRNA expression. In order to understand the impact of miR-205 on TCF21-induced inhibition of cell invasion, we co-transfected miR-205 mimic into ovarian cancer cells along with the TCF21 expression vector, and then measured invasion properties using Transwell invasion assay. We found that TCF21 alone inhibited the invasiveness of ovarian cancer cells. However, the transfection of miR-205 mimic into ovarian cancer cells diminished TCF21-mediated inhibition of cell invasion. Taken together, these findings indicate that miR-205 directly targets TCF21, and promotes cell invasion by repressing TCF21 expression in human ovarian epithelial carcinomas.

To better understand how the regulation of TCF21 by miR-205 affects invasion we investigated MMP expression. MMPs are expressed in nearly all tissue types and constitute a key group of proteinases for the regulation of cell-matrix composition. Their dysregulation is implicated in numerous pathological processes including embryogenesis, wound healing, inflammation, arthritis, cardiovascular diseases, and pulmonary diseases [40, 41]. MMPs are increased in the advanced stages of several cancers, and may be related to tumor progression and metastasis [42–44]. In women with ovarian cancer, elevated MMPs are an indication of tumor cell malignancy, and may be important for epithelial transformation and ovarian tumorigenesis [44]. We determined that the expression of MMPs was significantly increased in ovarian cancer tissue compared with normal ovarian tissues. We found that TCF21 decreased MMP-2 and MMP-10 expression in OVCAR-5, OVCAR-8 and SKOV-3 cells, and decreased MMP-9 expression in SKOV-3 cells. Furthermore, co-transfection of TCF21 expression plasmid with miR-205 blocked the effects of TCF21 on MMPs. These findings suggest that TCF21 may decrease ovarian cancer cell invasion properties by inhibiting the expression of specific MMPs. miR-205 targets TCF21 and regulates TCF21 mediated MMP expression in ovarian cancer cell invasion.

## Conclusions

We conclude that miR-205 regulates invasive behavior of epithelial ovarian cancer cells by targeting the tumor suppressor, TCF21. The results provide a new understanding of miR-205 and TCF21 expression and insight into the mechanisms in ovarian cancer tumorigenesis which could lead to the development of new anticancer therapies.

## Abbreviations

ECM: Extracellular matrix
miR: MicroRNA
MMP: Metalloproteinase
mRNA: Messenger RNA
TCF21: Transcription factor 21
